# Effects of Oxalic Acid on *Apis mellifera* (Hymenoptera: Apidae)

**DOI:** 10.3390/insects8030084

**Published:** 2017-08-07

**Authors:** Eva Rademacher, Marika Harz, Saskia Schneider

**Affiliations:** Institute of Biology/Neurobiology, Freie Universität Berlin, Königin-Luise-Str. 28-30, 14195 Berlin, Germany; marika.harz@lwk.nrw.de (M.H.); saskia.schneider@kabelmail.de (S.S.)

**Keywords:** *Apis mellifera*, *Varroa destructor*, oxalic acid dihydrate, toxicity, tolerance, sublethal effects

## Abstract

Oxalic acid dihydrate is used to treat varroosis of *Apis mellifera*. This study investigates lethal and sublethal effects of oxalic acid dihydrate on individually treated honeybees kept in cages under laboratory conditions as well as the distribution in the colony. After oral application, bee mortality occurred at relatively low concentrations (No Observed Adverse Effect Level (NOAEL) 50 µg/bee; Lowest Observed Adverse Effect Level (LOAEL) 75 µg/bee) compared to the dermal treatment (NOAEL 212.5 µg/bee; LOAEL 250 µg/bee). The dosage used in regular treatment via dermal application (circa 175 µg/bee) is below the LOAEL, referring to mortality derived in the laboratory. However, the treatment with oxalic acid dihydrate caused sublethal effects: This could be demonstrated in an increased responsiveness to water, decreased longevity and a reduction in pH-values in the digestive system and the hemolymph. The shift towards stronger acidity after treatment confirms that damage to the epithelial tissue and organs is likely to be caused by hyperacidity. The distribution of oxalic acid dihydrate within a colony was shown by macro-computed tomography; it was rapid and consistent. The increased density of the individual bee was continuous for at least 14 days after the treatment indicating the presence of oxalic acid dihydrate in the hive even long after a treatment.

## 1. Introduction

Oxalic acid dihydrate (OAD) is one of the most important organic acids used for the control of *Varroa destructor*. It has been known to be effective against the parasite since the end of the 20th century [1]. The European Group for Integrated Varroa Control developed OAD for the final application stage in beekeeping [2,3]. Three different application methods of OAD exist: trickling, spraying and evaporation. There are principal points to be considered concerning the medical treatment of honey bee colonies: the tolerability of the ingredient to bees, and the toxicity to mites, as well as its distribution in the colony, which again is directly affects the toxicity and efficacy of a substance. The trickling method of OAD combines high efficacy against *V. destructor* and low bee mortality. The tolerability in the bee colony has been documented in a concentration of 3.5% (*w*/*v*) OAD and a dose of 30–50 mL per colony for Central Europe [4]. Approval as a veterinary drug has been given in many countries worldwide over recent years, for Germany in 2006 [5].

So far, toxicological data on individual bees in the laboratory without combinatory effects has not been available. The mode of action in the colony was only partially clarified. In order to get a better understanding we tested the toxicity of OAD after dermal or oral application in the laboratory. Our aim was to define the no observed adverse effect level (NOAEL) and the lowest observed adverse effect levels (LOAEL) for OAD, including a safety margin for the dosage used in practical beekeeping. With this focus in mind, it is most important to look primarily at the dosage range of up to 10% bee mortality, as higher mortality rates are not acceptable for practical beekeeping. Therefore, we decided not to test very high dosages as it is unreasonable to kill large numbers of bees for non-relevant information. Furthermore, we wanted to understand which sublethal effects can be found and how OAD is distributed in the colony.

## 2. Materials and Methods

### 2.1. Laboratory Tests: Treatment of Individual Bees with OAD

#### 2.1.1. Investigation of Lethal Effects—Acute Oral and Dermal Toxicity

The toxicity tests were conducted during August and September using *Apis mellifera carnica* bees from our apiary at the Institute of Biology/Neurobiology, Berlin (Germany). The colonies were managed according to good beekeeping practice.

Worker bees were recruited from brood combs by brushing about 100 individuals into small cages. The test bees were approximately five to ten days old. The cages are made of wood (105/65/120 mm length/width/height) with bee wire and a glass plate on the sides. The caged bees were kept under laboratory conditions in the dark at 22 °C and 65% relative humidity, draft-free. The bees formed small clusters and were starved for 24 h to ensure an even distribution of food before being treated with OAD. After the application, they were held in small groups of 10 bees per cage and received food (Apifonda, Südzucker AG, Mannheim, Germany) and water *ad libitum*.

OAD (Caelo, Hilden, Germany) dissolved in sucrose solution (50% *w*/*w*) was applied to bees individually using two application forms: trickling 5 µL OAD solution onto the abdomen (dermal) or feeding 10 µL OAD solution (oral). Topical treatment with 5 µL was the maximum volume of solution applicable without considerable loss of agent. In feeding trials, 10 µL solution allows the feeding of even high dosages by lower concentrations, so they were well accepted by the bees. The test design for both treatments was identical. Each dosage was tested on 30 bees (three cages per dosage and ten bees in every cage). Each trial was replicated at least once leading to a minimum number of 60 bees tested for each dosage and treatment method. The control groups (three cages with ten bees per cage, one replicate) were treated in the same way but received only sucrose solution (50% *w*/*w*). The acute dermal toxicity test was conducted with different OAD concentrations: 3.5, 4.25, 5, 7.5 and 10% (*w*/*v*) and dosages: 175, 212.5, 250, 375 and 500 µg OAD/bee, respectively. The acute oral toxicity test consisted of concentrations respectively dosages: 0.1, 0.5, 0.75, 0.8 and 1% (*w*/*v*) corresponding to 10, 50, 75, 80 and 100 µg OAD/bee. Bee mortality data for test and control groups were collected 24, 48 and 72 h after the respective applications. Directly after the treatment, bees were observed for four hours and in time intervals up to 72 h for signs of behavioral changes.

#### 2.1.2. Investigation of Sublethal Effects

##### Responsiveness to Water and Ascending Concentrations of Sucrose Solution

The proboscis extension response (PER) was used to test bees’ responsiveness to water and ascending concentrations of sucrose solution (ACSS). Bees were recruited out of the colony as described for the previous experiments and individually marked. Bees were treated dermally with 5 µL of 3.5% OAD in sucrose solution (50% *w*/*w*) trickled onto the abdomen, the controls received sucrose solution only (n = 60). The proportion of animals releasing a PER was calculated for water and six concentrations of sucrose solution (0.1%, 0.3%, 1%, 3%, 10% and 30%). Each bee was tested twice: prior to the treatment and 24 h afterwards. Between tests the bees were kept in cages and starved for three hours prior to each test to ensure equal motivation. Solutions were applied to the antennae with a three-minute inter-trial interval. To ensure equal motivation, bees were starved for 3 h prior to the tests. Without anesthetization, every bee was carefully moved into a little plastic tube allowing only the head with antennae and proboscis to move freely. Bees were tested for water and then for sucrose responsiveness by applying first a drop of water followed by the ascending sucrose solutions to the antennae with a 3-min inter-trial interval. The proportion of animals releasing a PER was calculated.

##### Longevity under Laboratory Conditions

To obtain bees of the same age, a brood-frame was taken from a colony and put into an incubator at 34.5 °C. The hatched bees were removed daily. The young bees were kept in small cages and provided with food (Apifonda, Südzucker AG, Mannheim, Germany) and water *ad libitum*. Pretests showed a mortality rate of up to 60% when bees younger than five days were treated with OAD; for this experiment bees at the age of five days were chosen and treated dermally with 5 µL of 3.5% OAD in sucrose solution (50% *w*/*w*) trickled onto the abdomen or sucrose solution (controls), respectively (n = 50). Dead bees were counted and removed from the cages daily until the last bee had died. The test was repeated four times, a total of 200 bees per group were treated.

##### pH Values of the Digestive System and the Hemolymph

Worker bees were brushed from honey combs and individually treated with OAD. Dermal treatment was conducted with an amount of 5 µL 3.5% OAD in sucrose solution (50% *w*/*w*), dosage: 175 µg/bee, n = 120); oral treatment was performed with 10 µL 0.35% OAD in sucrose solution (50% *w*/*w*, dosage: 35 µg/bee, n = 120). The test animals were kept in cages as described above. In intervals of 24, 48 and 72 h post treatment the bees were frozen and subsequently dissected according to standard methods [6], the intestinal parts (crop, ventriculus and rectum) being removed and transferred onto micro-slides for pH measurement. An Inlab^®^ Surface Electrode, which enables the pH measurement of very small amounts of liquid (minimum of 5 µL), was carefully placed onto the different parts of the digestive system. The electrode was connected to a FiveGo^TM^ pH meter (limits of error: ±0.01 pH). The sampling of the hemolymph was conducted by the removal of the front and hind wings from the bee’s thorax. A slight pressure on the thorax enabled the extraction of the hemolymph from the wing base. The droplet of the hemolymph was absorbed with micro-capillaries and immediately transferred to micro-slides for pH measurement. Hemolymph samples (minimum of five bees per sample) were pooled to gain at least 5 µL of liquid.

### 2.2. Computer Tomography of Honey Bee Colonies: Distribution of OAD

Internal structures of a bee hive can be demonstrated by computed tomography [7]. Two honey bee nucleus colonies (*A. m. carnica*) were used for a distribution test with a macro-computed tomography scanner (macroCT). The colonies consisted of approximately 4000 individuals and had already formed a winter cluster. The treatments were conducted in November without brood. OAD (3.5% *w*/*v* in sucrose solution 50% *w*/*w*) was applied in the recommended dosage (according to package instructions for use-Oxuvar^®^) by trickling onto the bees in the bee space. OAD was mixed with the water-soluble contrast agent Unilux^®^ (Iopamidol, 370 mg iodine/mL) in a dosage of 185 µg/bee. The contrast agent Unilux showed no bee toxicity in a previous study [8].

**Figure 1:**
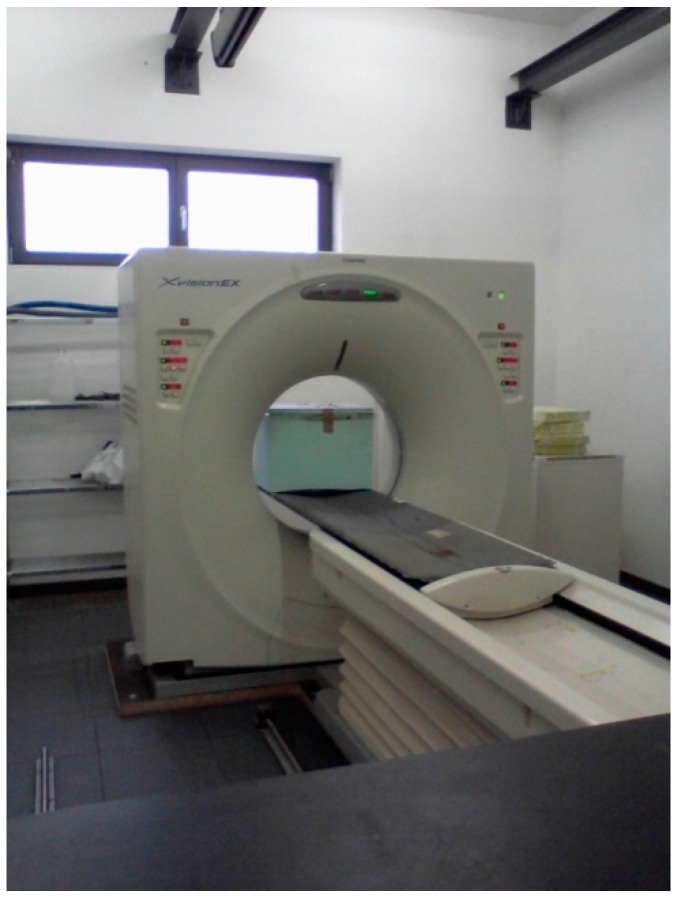
Bee colony placed in a macro-computed tomography scanner (macroCT).

For in-hive visualization of OAD distribution a macroCT scanner (Xvision, Toshiba) was used (Figure 1). With a helical scanner, a distance of 250 mm was examined ensuring the colony was captured completely. The CT images were reconstructed with a slice thickness of 2 mm (Table 1). For visualization via 2D images and data analysis we used the software eFilmTM LiteTM (MergeTM Healthcare 2008). The 2D images allowed the measuring of the density of individual bees in Hounsfield units (HU). This density is directly related to the amount of solution applied to the surface of the bee’s bodies. In a defined area of 100 cm^2^ in the central area of the comb, as well as in the boundary area of the bee cluster, the density of single bees (n = 144) was measured over three combs. In one colony, the measurements were conducted before application (control) and 10 min respectively thirty minutes after applying OAD. The second colony measurements were conducted before application (control) and 3, 7 and 14 days respectively after applying OAD (n ≤ 211/group). Only bees placed parallel to the macroCT sectional plane were quantified. The total dosage of radiation for the scan of the entire bee colony was 249.8 mGy, spread over the helix distance of 250 mm (125 slices of 2 mm thickness). Compared to the dosage of 500 mGy reported by [9] for biological effects in *Drosophila melanogaster*, this dosage can be considered harmless to bees.

### 2.3. Statistical Analysis

The statistical analysis was conducted using SigmaStat^®^ 3.0 software. Results were tested for differences in the bee mortality rate using the chi^2^-test on values at all-time intervals. The dose response curves obtained with SigmaPlot^®^ 3.0 software were the basis for the probit analysis and derivation of lethal dosage (LD) values. For the comparison of pH values of normally distributed data the t-test was used, alternatively in case of non-normally distributed data the Mann–Whitney rank sum test was used. The PER rates were compared between the groups for water responsiveness and within the groups for the sucrose solutions using chi²-test and McNemar’s test, respectively. The longevity data were analyzed using a Kaplan–Meier survival analysis, Gehan–Breslow. The density values of the bees in the small colony units were analyzed with the t-test. Regarding all statistical tests a difference was considered to be significant when the *p*-value obtained was lower than 0.05.

## 3. Results

### 3.1. Laboratory Tests: Treatment of Individual Bees with OAD

#### 3.1.1. Investigation of Lethal Effects—Acute Oral and Dermal Toxicity

**Figure 2:**
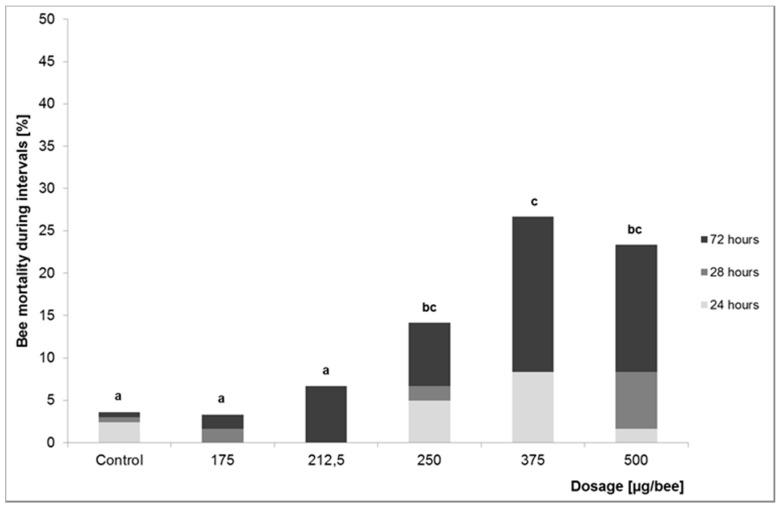
Bee mortality rates after dermal application of OAD during the three test intervals. Mortality rates with different lower-case letters are significantly different (chi^2^-test, *p* ≤ 0.05).

After dermal application of OAD the toxicity increased slowly during the observation time of 24 to 72 h. After 72 h the application of 175 and 212.5 µg/bee, respectively, showed no significant effect. After application of 250 µg OAD the bee mortality was significantly higher (chi^2^-test, *p* = 0.003) than at 175 and 212.5 µg/bee. In the dosages 375 and 500 µg/bee the mortality increased to >20% (Figure 2). The NOAEL (72 h) for dermal application was 212.5, the LOAEL 250 µg OAD/bee. The extrapolated LD_10_ (72 h) is 256.4 µg OAD/bee. The LD_10_ 48 h after application reached a higher value than after 72 h: 467.7 µg/bee. The LD_10_ 24 h after application exceeded this value but could not be determined from the data (Table 2).

**Figure 3:**
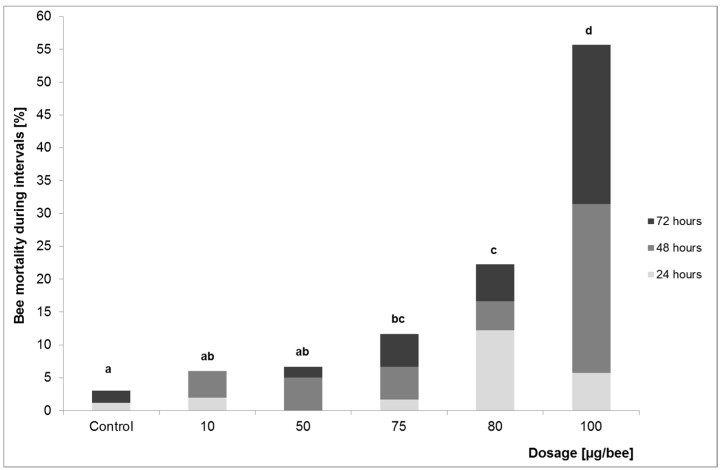
Bee mortality rates after oral application of OAD during the three test intervals. Mortality rates with different lower-case letters are significantly different (chi^2^-test, *p* ≤ 0.05).

After oral application, bee mortality occurred at relatively low concentrations compared to the dermal treatment (Figure 3). 10 and 50 µg did not cause a mortality rate significantly different from the control group, while 75 µg resulted in significantly higher bee mortality (chi^2^-test, *p* = 0.027). A total of 100 µg killed 55% of treated animals after 72 h. The NOAEL (72 h) for oral application was 50, the LOAEL was 75 µg OAD/bee. The LD_10_ obtained by probit analysis was 60.3 µg/bee. The LD_10_ 48 h after application reached higher values than after 72 h: 68.1 µg/bee. The LD_10_ 24 h after application was expected to exceed this value but this could not be determined from the data (Table 2).

During the experiment, changes in behavior were observed only after oral application of dosages also causing increased mortality within this period (≥75 µg/bee). The bees were less active, showed minor movement and slowly formed bee clusters at the top of the cage. Shortly after the application they showed increased self-grooming. Some bees also extended the proboscis.

#### 3.1.2. Investigation of Sublethal Effects

##### Responsiveness to Water and Ascending Concentrations of Sucrose Solution

**Figure 4:**
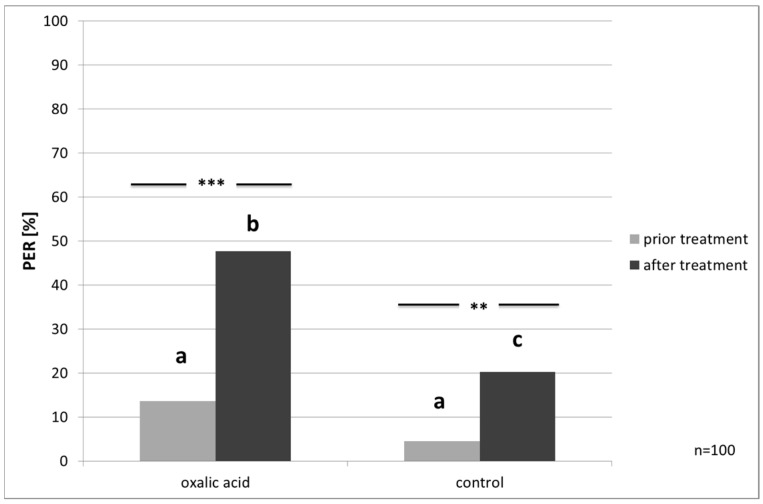
PER (Proboscis Extension Response) to water prior and after treatment: both groups show increased responsiveness after the treatment (McNemar’s test, *p* ≤ 0.001 and *p* = 0.004,). Different lower-case letters indicate significant differences between groups, before and after treatment (chi²-test, *p* ≤ 0.001) additionally; significant differences are marked with asterisks’.

The PER on water increased after the treatment in the test (McNemar’s test, *p* ≤ 0.001) and control bees (McNemar’s test, *p* ≤ 0.01, Figure 4). However, bees treated with OAD showed significant higher response rates to water than the controls (chi²-test, *p* ≤ 0.001).

**Figure 5:**
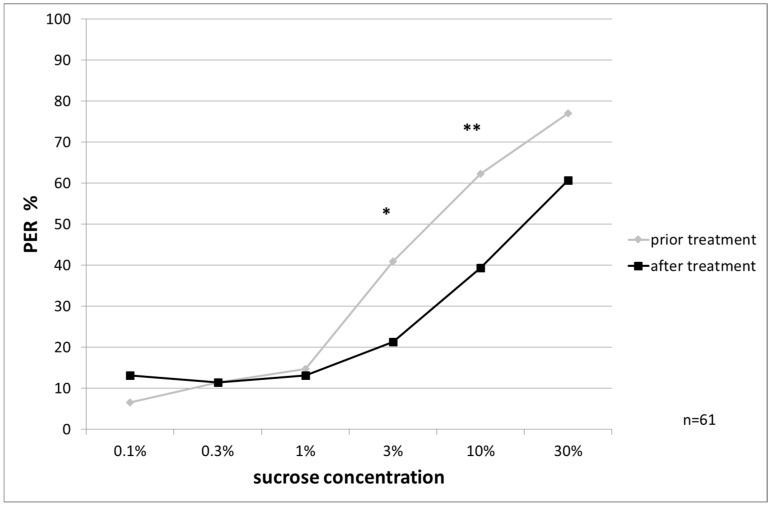
PER to ascending sucrose concentrations from control bees: response is significantly decreased at 3% and 10% concentration (McNemar’s test, *p* = 0.025 and *p* = 0.018), significant differences are marked with asterisks’.

The sucrose responsiveness was also influenced by treatments, in the control group the responsiveness decreased significantly at 3% and 10% concentration (McNemar’s test, *p* ≤ 0.025, Figure 5).

**Figure 6:**
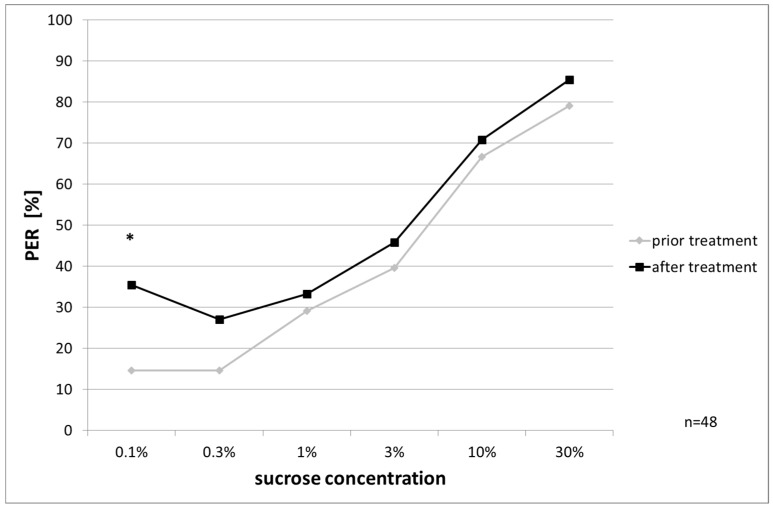
PER to ascending sucrose concentrations from OAD-treated bees: response after treatment is significantly increased at 0.1% concentration (McNemar’s test, *p* = 0.024), significant differences are marked with asterisks’.

Bees treated with OAD showed increased sucrose responsiveness, significantly at 0.1% (McNemar’s test, *p* = 0.024, Figure 6).

##### Longevity under Laboratory Conditions

**Figure 7:**
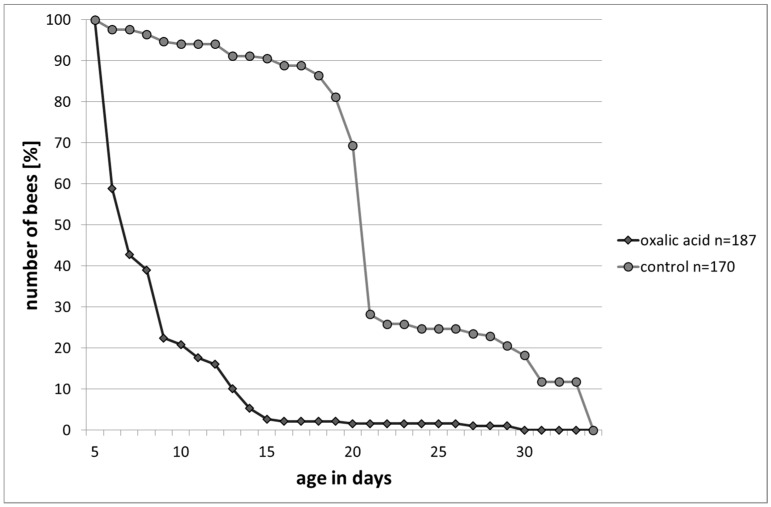
Survival curves under laboratory conditions: bees in the control group survived significantly longer than bees treated with OAD (Kaplan–Meier survival analysis, Gehan–Breslow, *p* ≤ 0.001).

The highest proportional bee mortality occurred directly after the treatment (test group) and between the 20th and 21st day after hatching (control group). In the test group bees lived at least for two days, for a maximum of 31 days and on average for 6.4 days. The bees in the control group lived for an average of 24.4 days (min. two days, max. 33 days). This can be demonstrated in the trend of the survival curves (Kaplan–Meier survival analysis, Gehan–Breslow, *p* ≤ 0.001, Figure 7).

##### pH Values of the Digestive System and the Hemolymph

**Figure 8:**
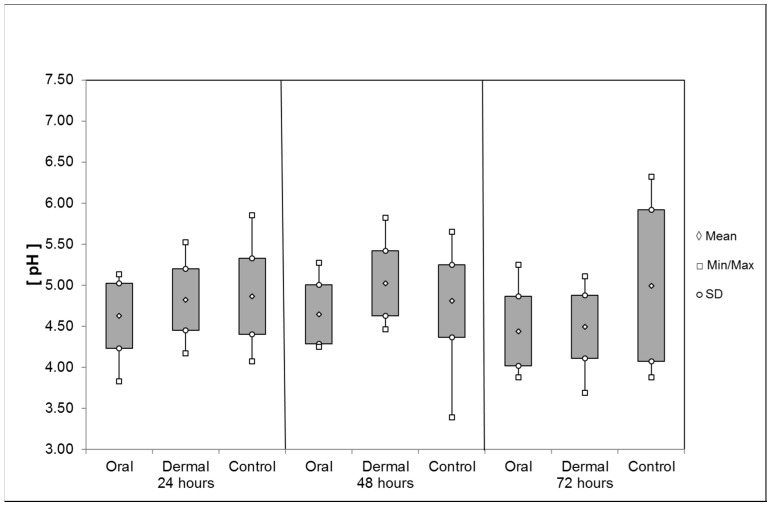
Average pH of the liquid contents of the honeybee crop after oral and dermal treatment (n ≤ 52/group).

*Crop*: The pH values of the crops’ content after *oral* OAD application were subject to slight variation (Figure 8). The average pH value (±SD) after oral application of OAD (24 h: 4.62 ± 0.4; 48 h: 4.64 ± 0.36; 72 h: 4.44 ± 0.43) was lower during 72 h than the control. After the *dermal* application, the pH values were at 24 h: 4.82 ± 0.37 and 48 h: 5.02 ± 0.4 remaining at the control level. Only during the last interval did the pH subside to 4.49 ± 0.38 (72 h). The pH values obtained by the control group were 24 h: 4.86 ± 0.46; 48 h: 4.80 ± 0.44; and 72 h: 4.99 ± 0.92. However the differences were not significant (Mann–Whitney rank sum test, *p* ≥ 0.05), and the standard deviation of means is comparatively high.

**Figure 9:**
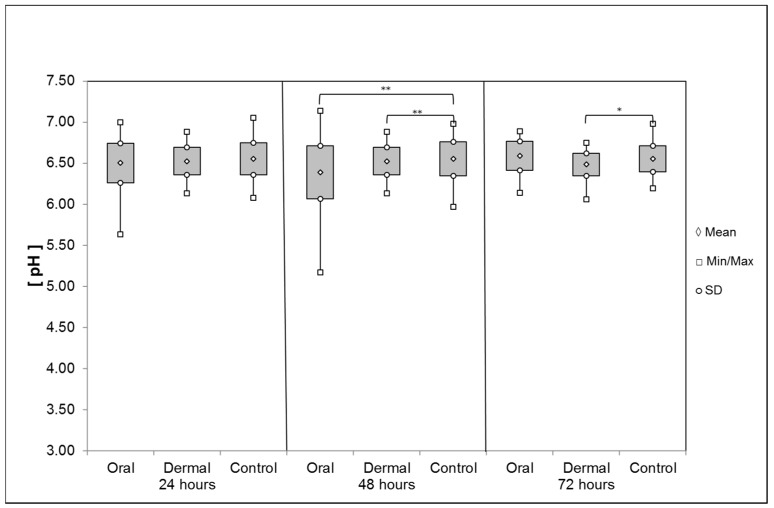
Average pH of the ventriculus contents after oral and dermal treatment (n ≤ 68/group). Significant differences are indicated by asterisks (48 h: Mann–Whitney rank sum test, *p* = 0.002; 72 h: t-test, *p* < 0.05); significant differences are marked with asterisks’.

*Ventriculus*: The average pH values of the liquid ventriculus contents were significantly reduced between 24 and 48 h after *oral* application of OAD: 6.50 ± 0.24 (24 h) and 6.38 ± 0.32 (48 h, Mann-Whitney-rank-sum-test, *p* = 0.002), respectively (Figure 9). During the last interval, the pH value averaged 6.59 ± 0.17 (72 h). After the *dermal* application of OAD, the pH value was on average 6.52 ± 0.17 (24 h). Within the following intervals the pH reduced significantly to 6.40 ± 0.21 (48 h) compared with the control group (t-test, *p* = 0.002) and 6.48 ± 0.14 after 72 h (t-test, *p* = 0.028). The pH values obtained by the corresponding control groups remained at a constant level: 24 h: 6.55 ± 0.2; 48 h: 6.55 ± 0.21; 72 h: 6.55 ± 0.16.

**Figure 10:**
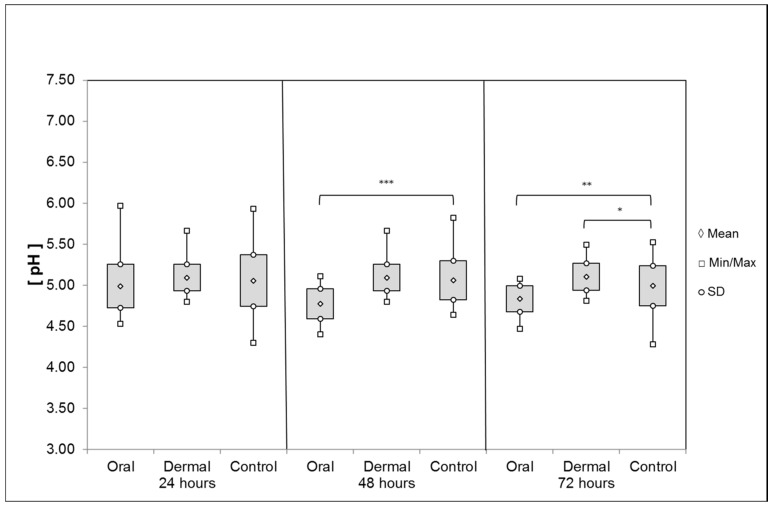
Average pH of the rectum contents after oral and dermal treatment (n ≤ 68/group). Significant differences are indicated by asterisks (48 h: t-test, *p* = 0.001; 72 h: t-test, *p* < 0.05); significant differences are marked with asterisks’.

*Rectum*: The pH value of the liquid rectum contents averaged 4.98 ± 0.27 24 h after *oral* OAD application (Figure 10). After 48 h, the pH was significantly reduced to 4.77 ± 018 (t-test, *p* < 0.001); after 72 h, the pH reached 4.83 ± 0.16 and remained significantly different to the control group (t-test, *p* = 0.003). After *dermal* application of OAD, the pH value averaged 5.09 ± 0.16 (24 h) and 5.11 ± 0.18 (48 h). During the last interval, the pH was 5.10 ± 0.17 and significantly different to the control group value (t-test, *p* = 0.016). The pH values obtained by the corresponding control groups were: 24 h: 5.05 ± 0.31; 48 h: 5.05 ± 0.24; 72 h: 4.99 ± 0.24.

**Figure 11:**
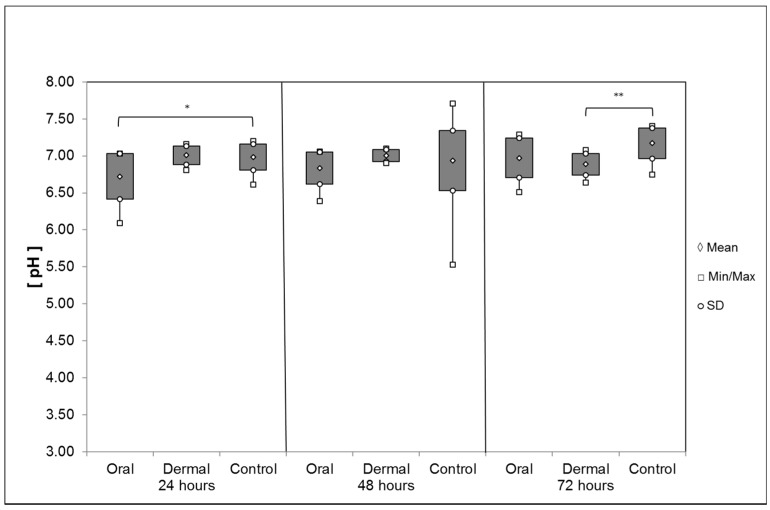
Average pH of the hemolymph after oral and dermal treatment (n ≤ 26/group). Significant differences are indicated by asterisks’ (24 h: Mann–Whitney rank sum test, *p* = 0.011; 72 h: t-test, *p* = 0.006); significant differences are marked with asterisks’.

*Hemolymph*: OAD, 24 h after *oral* application, caused a significant reduction in the average pH value of the hemolymph with 6.72 ± 0.31 compared to the control group (6.98 ± 0.17; Mann–Whitney rank sum test, *p* = 0.011, Figure 11). During the remaining intervals, the pH increased to 6.83 ± 0.22 (48 h) and 6.97 ± 0.27 (72 h). After *dermal* application, the pH values measured at the first and second intervals were 7.00 ± 0.12 (24 h) and 7.00 ± 0.08 (48 h), respectively, and thus similar to the control groups: 6.98 ± 0.17 (24 h); 6.96 ± 0.26 (48 h). After 72 h, the average pH value was 6.88 ± 0.15, significantly reduced in comparison to the control group 7.17 ± 0.21 (t-test, *p* = 0.006).

### 3.2. Computer Tomography of Honey Bee Colonies: Distribution of OAD

**Figure 12:**
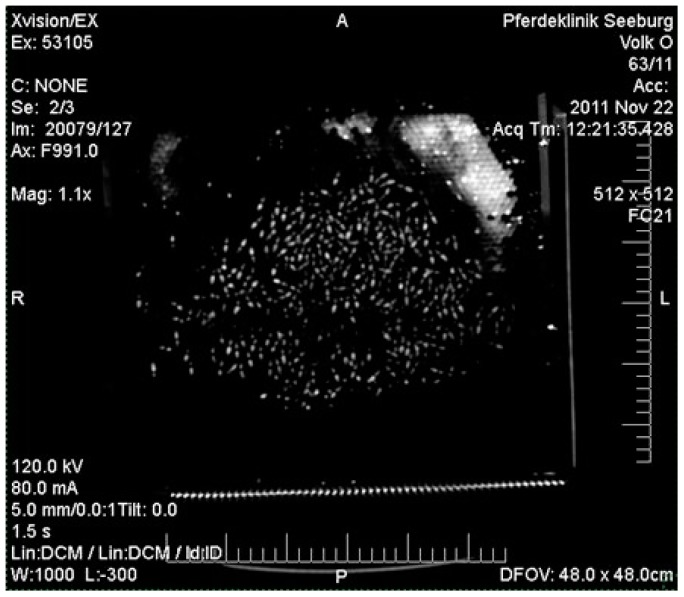
MacroCT scanner image: bees on the comb, 30 min after application of OAD and Unilux. Visualization of a comb with bees sitting on it: dark areas show low densities and bright areas show high densities.

**Figure 13:**
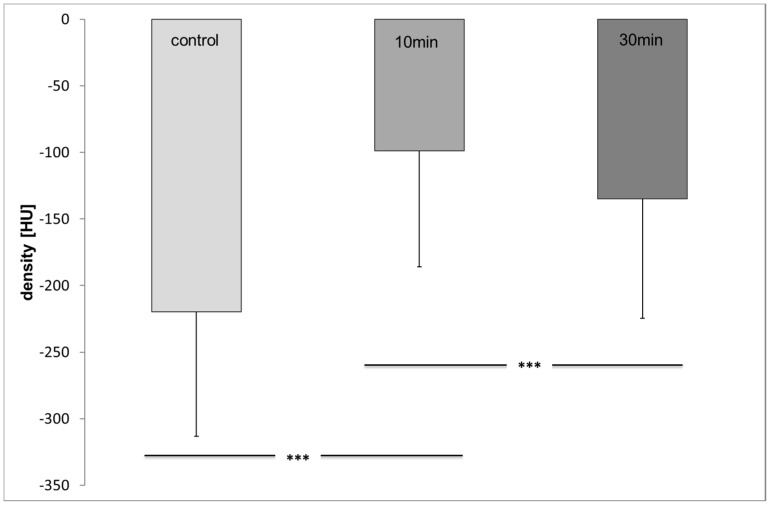
Density values before (control) and 10 and 30 min after treatment (n ≤ 144/group; t-test, *p* ≤ 0.001), significant differences are marked with asterisks’.

The distribution of OAD in the colony after topical application was demonstrated by macroCT scanning (Figure 12). The control measurements achieved a mean density value of −219.77 ± 93.3 HU. The relatively high standard deviation was caused by the heterogeneity of the bees’ body mass. After application of OAD (10 min) the density value increased to a mean value of −98.97 ± 87.06 HU, which was significantly different from the controls (t-test, *p* ≤ 0.001). Thirty minutes after application the mean value was −134.98 ± 89.5 HU, significantly different compared to the controls and the values achieved after 10 min (t-test, *p* ≤ 0.001, Figure 13).

**Figure 14:**
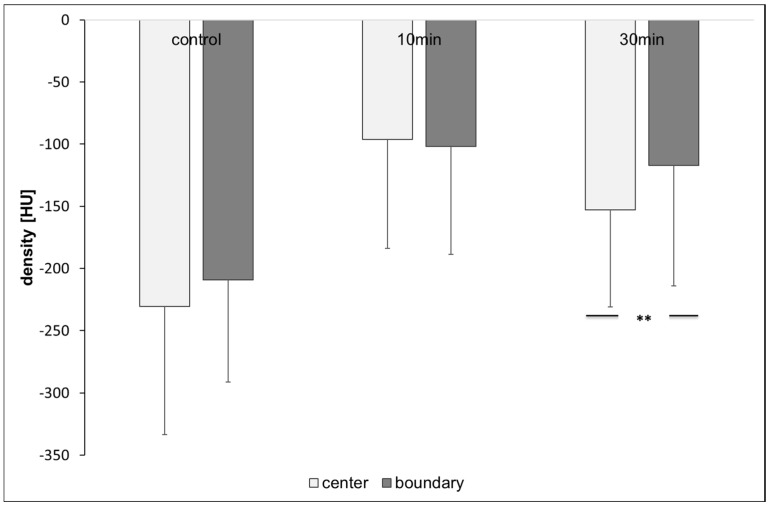
Density values of the comb areas center and boundary: significant differences between areas occur 30 min after treatment (n ≤ 72/group; t-test, *p* = 0.015), significant differences are marked with asterisks’.

The mean value (−96.03 ± 87 HU) in the central area of the combs 10 min after treatment of the bees was comparable to the bees in the boundary area (−101.92 ± 86.7 HU). However, the mean density values thirty min after the treatment were significantly different compared to the central area (−153.04 ± 77.8) and boundary area (−116.9 ± 97.1) of the combs (t-test, *p* = 0.015, Figure 14).

**Figure 15:**
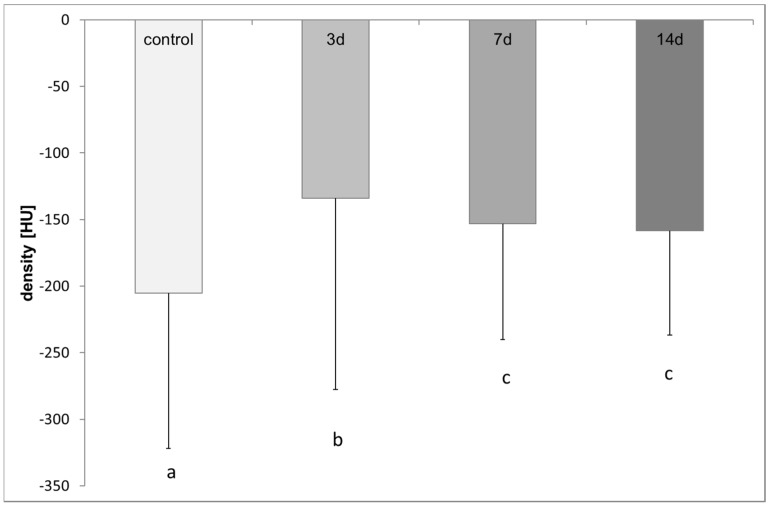
Density values before (control) and 3, 7 and 14 days after treatment: different lower-case letters indicate significant differences (n ≤ 211/group; t-test, *p* ≤ 0.001).

In the colony examined for the long-term distribution, the control measurements achieved a mean density value of −200.61 ± 86.87 HU. Three days after the application of OAD the density value increased to a mean value of −128.1 ± 89.92 HU and was significantly different from the control measurement (t-test, *p* ≤ 0.001, Figure 15). Seven and 14 days after application the mean value was −151.61 ± 77.14 HU and −158.46 ± 78.32 HU, respectively, significantly different compared to the controls and the values achieved after three days (t-test, *p* ≤ 0.001, Figure 15).

## 4. Discussion

We found unequal results of OAD toxicity after dermal and oral application—ingestion of OAD is much more toxic to bees than application on the cuticula, which was relatively well tolerated. This corresponds with our first report of bee tolerability concerning OAD as a single compound [10].

Investigations of toxicity are often conducted with combined substances. This makes it difficult to compare these results with our findings. Concerning the dermal application of the toxic effects of different dosages are reported in the literature. Dosages <100 µg/bee did not cause significant mortality after 48 h, this is less than half the dosage of 212.5 µg/bee with no observed mortality in our test. The described LD_10_ (48 h) of 176.68 µg/bee was derived from a combinatory effect of OAD and acetone on anaesthetized worker bees (CO_2_) [11]. It was lower than the LD_10_ (48 h) of OAD as a single agent in our trial, which reached a level of 467.7 µg/bee. A combinatory effect of OAD and acetone on anaesthetized worker bees (CO_2_) may explain the explicitly higher responsiveness of the tested animals in the cited trials compared to our findings. Other tests with a polyhybrid subspecies of *A. mellifera* resulted in a level without observed effect of 400 µg/bee and a lowest observed effect level of 600 µg/bee after 48 h [12].

In our experiments 175 µg/bee, corresponding to the 3.5% solution (30–50 mL depending on colony size) used in beekeeping practice, did not cause mortality for individual bees, different from controls 72 h after dermal application. Oral application resulted in high bee mortality at relatively low concentrations compared to the dermal treatment. Bees reacted much more sensitively to the oral application of OAD. The LOAEL for dermal application is higher than for oral application by a factor >3.

To assess risk of an applied substance, not only mortality but also the effects on physiological processes and behavior after pesticide exposition must be considered as described [13]. Physiological effects concerning acetylcholinesterase and glutathione S-transferase activities, as described for exposure to fluvalinate, are not documented after OAD treatment (3% in 32% sucrose solution *w*/*w*, 50 mL/colony) on pupae, newly emerged, nursery and forager bees [14,15]. Honey bee larvae, treated with OAD by spraying (about 121 µg solution/larvae) undergo histologic changes—accidental cell death leading to necrosis [16]. These effects may be causally responsible for the brood death after application of OAD (3% in 50% sucrose solution, two treatments during summer) in breeding colonies [17]. In beekeeping practice, acute damages to the brood can be excluded when honeybee colonies are treated with OAD during the brood free period. However, long term effects are possible; OAD has been found in bee colonies even six months after topical application following regular treatment [18]. Long-term effects (up to four months after application) like a reduced amount of brood in treated colonies (3% OAD *w*/*v*, 4 mL per comb side, spraying) have been reported [19].

After dermal and oral application, respectively, of high dosages up to 1320 µg/bee, OAD was recovered in the internal organs and the hemolymph of adult bees [20,21]. It is assumed that after dermal application the cuticula can be penetrated by the acid [21]. Also, pathological repercussions e.g., degeneration of rectal epithelium, malphigian tubules and ventriculus, have been described after dermal application [20]. C^14^-marked OAD could be found in the abdominal structures of worker bees after topical application into the colony [22].

The treatment with OAD caused sublethal effects on *A. mellifera*. First indications of changes in the pH of internal organs and the hemolymph after a single dermal application of OAD with a dosage of 175 µg/bee have been provided [23]. The individual treatment of honeybees with OAD in our experiments changed the pH-value of the intestinal parts and the hemolymph. The pH values of the crop contents reflect the acid application, specifically after oral ingestion. Significant differences could be found in the pH values of the ventriculus 48 h after oral and 72 h after dermal treatment: These deferred reductions in the tested time intervals could be caused by the slower penetration of the acid through the cuticula compared to the direct oral intake. Differences in the pH structure of the rectum and the hemolymph were also found, the pH reduction occurs faster after oral application. The proof of a pH reduction in the digestive system even after 72 h indicates a long disposition of OAD in the bee. The returned balance of the pH measured in the hemolymph 48 h after oral application also indicates a possible buffer capacity of the hemolymph.

We could demonstrate that OAD moving through the digestive system or penetrating the cuticula modified the pH structure of the honeybee’s intestinal parts and the hemolymph. The shift towards stronger acidity after OAD treatment supports that damage to the epithelial tissue and organs [20] may be caused by hyperacidity. It also corresponds to the timescale of recovered OAD after oral application [21]. The increased acidity can cause chemical burns which eventually lead to necrosis [16], however a threshold cannot be derived from our tests due to the low dose applied.

Further sublethal effects could be demonstrated in a decreased longevity under laboratory conditions and increased responsiveness to water. Bees treated with OAD died much sooner than bees treated with sucrose solution. The increased responsiveness to water 24 h after OAD treatment indicated an acidosis of the bees, which they may compensate with an increased uptake of water. This assumption is supported by the shift towards stronger acidity, found—after OAD treatment conducted—in the measurements of the pH-values. These results indicate a general impairment of the bees after treatment. The treatment in autumn/winter affected primarily long-living winter bees which are essential for winter survival and successful colony development in the spring. Treatment during the summer with brood can lead to substantial brood damage [15] as described above. Even by treating artificial swarms or nucleus colonies, it cannot be certain that damages will not occur due to the long-term exposure to OAD in the colony [18,19].

It has been described that after dermal administration bees carry white deposits primarily on the body [11,20] and later also on the third pair of legs [24]. Concerning sublethal effects in the colony after individual dermal application of 175 µg/bee, changes in behavior were found [24]: the bees were less active, showed reduced brood care, increased grooming behavior, and they also had a reduced life span.

In beekeeping practice OAD is mostly applied topically. The distribution in the colony may occur on two pathways: (1) through oral intake and distribution via trophallaxis; and/or (2) contact between bees and also contaminated hive material. OAD vapors in the colony seem to be of minor importance as OAD has a low volatility [25]. However, the colony treatment using 3.5% OAD solution (30–50 mL per colony, depending on colony strength, applied by trickling) corresponds to an individual dosage of approximately 175 µg OAD/bee. This dosage is well tolerated by individual bees in the laboratory and is well below the lowest observed adverse effect level concerning mortality after dermal application in our test.

OAD applied orally in the laboratory causes high bee mortality. As the mortality observed in the treated colonies was not highly increased, OAD was probably ingested in relatively low amounts by grooming and/or trophallaxis. The oral intake of OAD after treatment at the colony level seems to be of little importance. The threshold value derived in the laboratory was therefore not exceeded for most of the target animals. This corresponds with the findings that when OAD is applied in a solution with high sugar content the bee mortality increases [26]. This suggests that high sugar content leads to more ingestion. In laboratory trials, significantly higher bee mortality was found when sugar was added to the OAD solution [27].

In practical beekeeping, appropriate use of OAD (on average 175 µg/bee) by topical application in the field is relatively safe for *A. mellifera* on the colony level, even when some individuals die. Based on our findings in the laboratory, the threshold with first adverse effects (LOAEL) could be reached in the colony when an overdose of 43% for the individual bee is applied. However, due to the attractiveness of sucrose solutions to bees they ingest the solution even if it contains toxic substances. A sugar substitute e.g., glycerol, with a high viscosity and desirable distribution can prohibit oral uptake by the bees [9]. It could optimize the application of OAD, but so far it has not been approved as a veterinary drug ingredient for bees in combination with OAD.

High acaricidal efficacy after topical application was only found when bee to bee contact took place [11]. In further trials, when body contact between the bees was prevented, but trophallaxis allowed, it has been shown that trophallactic interaction did not lead to mite mortality; bee to bee contact seemed to be the primary route of distribution of OAD in acaricidal relevant dosages [28].

Due to only minor oral intake, systemic efficacy against *V. destructor* seems to be improbable. A systemic effect requires a transfer of the acid into the hemolymph of the bee. OAD was found in the hemolymph only after application of very high dosages [20,21]. After application of the 3.5% OAD solution (175 µg/bee), as used in beekeeping, changes in the pH structure were found, but OAD was not recovered in the hemolymph of single bees at a detection limit of 2.5 µg [24]. This amount used in beekeeping practice seems to be too low to reach a systemic toxicity to *V. destructor* through ingestion. Therefore, we conclude that the mode of action in the colony must be contact poisoning against *V. destructor*.

In order to reach high efficacy, the ingredient acting by contact must be distributed in the colony. The distribution of OAD was shown by macroCT. The results of the roentgenoscopy showed high density values for the individual bees in the test, much higher than in the control measurement. A good distribution was already achieved after 10 min; this could be documented in the central and boundary areas of the combs. Lower density values in the central comb areas compared to the boundary regions obtained after thirty minutes reflected the movement of the bees. After thirty minutes the density was generally lower, which led us to the assumption that OAD was now also spread to the material, e.g., the wall of the hive. Bees have constant contact with the hive material; therefore, OAD can be distributed again onto the bees, maintaining a long-term contact with the acid. OAD on hive material can be found even several months after application [18]. The macroCT analysis demonstrated a rapid and consistent distribution of OAD involving a reduction of the individual dosage over time. Even after 14 days, the density of the bees was still significantly higher than prior to treatment, indicating a potential efficacy of at least up to 14 days. The results from the field trials, where the maximum efficacy against mites was reached ten days after treatment, support this assumption [29].

## 5. Conclusions

OAD used to treat varroosis of *A. mellifera* shows a rapid and consistent distribution in the colony for at least up to 14 days, and high efficacy against the mite, but also lethal and sublethal effects. In practical beekeeping, appropriate use of OAD (one topical application, on average 175 µg/bee) is relatively safe for *A. mellifera* at the colony level, even when some individuals are lost. However, ingestion leads to high mortality. The reported sublethal effects are highly decreased longevity, a reduction in pH-values in the digestive system and the hemolymph, and an increased responsiveness to water. The shift towards stronger acidity after treatment confirms that damage to the epithelial tissue and organs is likely to be caused by hyperacidity. Pathological repercussions e.g., degeneration of rectal epithelium, malpighian tubules and ventriculus may also occur.

These results indicate a general impairment of the bees after treatment. The treatment in autumn or winter affects primarily long-living winter bees which are essential for winter survival and successful colony development in the spring. Treatment during summer with brood can cause substantial brood damage. Even when treating artificial swarms or nucleus colonies it cannot be certain that damages will not occur due to the extensive exposure to OAD in the colony. Long-term effects such as reduced amount of brood in treated colonies have been reported.

OAD is one of the most important organic acids used for the control of *V. destructor*. It is indispensable but must be dosed precisely and applied as seldom as possible to prevent sublethal damages which eventually lead to the loss of bees. Long disposition in the bee hive can cause accumulation of the acid and therefore induce further damage.

## Figures and Tables

**Table 1:** Technical data for *A. mellifera* colony scanning.

Parameter	Resolution
Slice thickness	2 mm
Pitch	2.5
Helix	250 mm
Peak X ray voltage	120 kV
X ray tube current	80 mA
Total scan time	75 s
Matrix	512x512
Scan field of view	large
Display field of view	480 mm
Window width	1000
Window level	−300
Total scan dosage	249.8 mGy

**Table 2:** Toxicity parameters after dermal and oral application of OAD.

	Dermal Application (µg/bee)		Oral Application (µg/bee)	
	48 h	72 h	48 h	72 h
LD_10_	467.7	256.4	68.1	60.3
NOAEL	n.d. ^b^	212.5	75	50
LOAEL ^a^	n.d. ^b^	250	80	75
chi^2^-test		*p* = 0.003	*p* < 0.001	*p* = 0.027

^a^ These values represent the lowest dosage with significant difference in bee morality compared to controls; ^b^ n.d. not defined, no significant differences occurred after 48 h; *p*-values refers to the first significant increase in mortality (LOAEL).

## References

[1] Popov E.T., Melnik V.N., Matchinev A.N. (1989). Application of oxalic acid in varroatosis. XXXII International Congress Apimondia.

[2] Nanetti A., Büchler R., Charriere J.D., Fries I., Helland S., Imdorf A., Korpela S., Kristiansen P. (2003). Oxalic acid treatments for varroa control (review). Apiacta.

[3] Rademacher E., Imdorf A. (2004). Legalization of the use of oxalic acid in Varroa control. Bee World.

[4] Rademacher E., Harz M. (2006). Oxalic acid for the control of Varroosis in honey bee colonies—A review. Apidologie.

[5] Rademacher E. (2006). Oxalsäure als Tierarzneimittel zur Bekämpfung der Varroose legal einsetzbar. ADIZ/die biene/Imkerfreund.

[6] Dade H.A. (1977). Anatomy and Dissection of the Honeybee.

[7] Greco M.K. (2010). Imaging techniques for improved bee management. ALP Sci..

[8] Rademacher E., Fahlberg A., Raddatz M., Schneider S., Voigt K. (2013). Galenics: Studies of the toxicity and distribution of sugar substitutes on *Apis mellifera*. Apidologie.

[9] Kanao T., Okamoto T., Miyachi Y., Nohara N. (2003). Parental exposure to low dose X-rays in *Drosophila melanogaster* includes early emergence in offspring, which can be modulated by transplantation of polar cytoplasm. Mutat. Res..

[10] Rademacher E., Harz M. (2009). Oxalic acid: Toxicology on *Apis mellifera*. Apidologie.

[11] Aliano N.P., Ellis M.D., Siegfried B.D. (2006). Acute contact toxicity of oxalic acid to *Varroa destructor* (Acari: Varroidae) and their *Apis mellifera* (Hymenoptera: Apidae) hosts in laboratory bioassays. J. Econ. Entomol..

[12] Carrasco-Letelier L., Mendoza Y., Ramallo G. (2012). Acute contact toxicity of oxalic acid on honeybees in the southwestern zone of Uruguay. Chil. J. Agric. Res..

[13] Desneux N., Decourtye A., Delpuch J.M. (2007). The sublethal effects of pesticides on beneficial arthropods. Annu. Rev. Entomol..

[14] Rouibi A., Bouchema W., Loucif-Ayad W., Achou M., Soltani N. (2016). Risks assessment of two acaricides (fluvalinate ad oalic acid) in *Apis mellifera intermissa* (Hymenoptera, Apidae): Acethylcholinestease and glutathione S-trasferase activities. J. Entomol. Zool. Stud..

[15] Brosgaard C.J., Jensen S.E., Hansen C.W., Hansen H. (1999). Spring treatment with oxalic acid in honeybee colonies as Varroa control. DIAS Rep. Hortic..

[16] Gregorc A., Pogaènik A., Bowen I.D. (2004). Cell death in honeybee (*Apis mellifera*) larvae treated with oxalic acid. Apidologie.

[17] Hatjina F., Haristos L. (2005). Indirect effects of oxalic acid administration by trickling method on bee brood. J. Apicult. Res..

[18] Moosbeckhofer R., Rademacher E. (2012). Personal communication.

[19] Higes M., Meana A., Suarez M., Llorente J. (1999). Negative long-term effects on bee colonies treated with oxalic acid against *Varroa jacobsoni* Oud. Apidologie.

[20] Martin-Hernandez R., Higes M., Perez J.L., Nozal M.J., Gomez L., Meana A. (2007). Short term negative effect of oxalic acid in *Apis mellifera iberiensis*. Span. J. Agric. Res..

[21] Nozal M.J., Bernal J., Gomez L., Higes M., Meana A. (2003). Determination of oxalic acid and other organic acids in honey and in some anatomic structures of bees. Apidologie.

[22] Nanetti A., Ghini S., Gattavecchia E., Bartolomei P., Marcazzan G.L., Massi S. (2003). Pharmacodynamics of Oxalic Acid and Treatment Residues in Honey.

[23] Raddatz M., Rademacher E. (2010). Sublethal effects of oxalic acid on *Apis mellifera* L. (Hymenoptera: Apidae): Pharmacodynamics. Apidologie.

[24] Schneider S., Eisenhardt D., Rademacher E. (2012). Sublethal effects of oxalic acid on *Apis mellifera* L. (Hymenoptera Apidae): Changes in behavior and longevity. Apidologie.

[25] O’Neil M.J. (2001). The Merck Index: An Encyclopedia of Chemicals, Drugs and Biologicals.

[26] Charriere J.D., Imdorf A. (2002). Oxalic acid treatment by trickling against *Varroa destructor*: Recommendations for use in Central Europe and under temperate climate conditions. Bee World.

[27] Toomemaa K., Martin A., Williams I. (2010). The effect of different concentrations of oxalic acid in aquaeous and sucrose solution on Varroa mites and honey bees. Apidologie.

[28] Aliano N.P., Ellis M.D. (2008). Bee-to-bee contact drives oxalic acid distribution in honey bee colonies. Apidologie.

[29] Rademacher E., Harz M., Schneider S. (2015). The development of HopGuard^®^ as a winter treatment against *Varroa destructor* in colonies of *Apis mellifera*. Apidologie.

